# Supplementary material for: Electromechanics in MoS2 and WS2: nanotubes vs. monolayers
Source: Sci Rep. 2013 Oct 16;3:2961. doi: 10.1038/srep02961 (PMC3797429; doi:10.1038/srep02961)
Supplement: Supplementary Information [file srep02961-s1.pdf]

Electromechanics in MoS_2 and WS_2: nanotubes vs. monolayers
Supporting Information

Mahdi Ghorbani-Asl,[1] Nourdine Zibouche,[1,2] Mohammad Wahiduzzaman,[1]
Augusto F. Oliveira,[1,2] Agnieszka Kuc,[1] & Thomas Heine[1*]
[1]*School of Engineering and Science, Jacobs University Bremen,*
Campus Ring 1, 28759 Bremen, Germany,
[2]*Scientific Computing & Modelling NV, Vrije Universiteit,*
De Boelelaan 1083, 1081 HV Amsterdam, The Netherlands

TABLE S1: Calculated Raman and IR phonon active modes, their symmetry representations, and the average slope of the linear fitting to the calculated phonon frequencies versus tensile strain of the (n,n) NTs (see the main text). The numbers for the (n,0) NTs are given in parenthesis.

**Symmetry/Activity**				**MoS_2**		**WS_2**	
**MW/DW**		**SW**		ε = **0–5%**	ε = **5–10%**	ε = **0–5%**	ε = **5–10%**
E_{2_g}	R	E'	IR+R	1.6 (1.9)	2.4 (3.3)	1.6 (1.7)	2.9 (3.7)
A_{1_g}	R	A'	R	1.4 (1.3)	1.1 (1.4)	1.1 (1.9)	2.7 (2.6)

The Slater-Koster parameters for the DFTB calculations have been optimized based on the electronic band structures calculated for bcc, fcc, and simple cubic lattices of Mo, W, and S, as well as hexagonal lattices of bulk and monolayer models of TMDs. The reference band structures were calculated with all-electron DFT using PBE density functional[1] and TZP (tripple-ζ with one polarization function) basis set and relativistic ZORA correction, as implemented in ADF/BAND code.[2–4]

Since monoatomic properties also influence the quality of the Slater-Koster tables, we have considered two electronic configuration for Mo. The experimental one (Mo: [Kr] $4d^5$ $5s^1$) did not result in a good description of the top valence bands and lower conduction bands. Therefore, we have chosen an electronic configuration based on the Pauli diagram, i.e. Mo: [Kr] $4d^4$ $5s^2$. The latter configuration results in slightly larger band gaps, but other features,

*Electronic address: t.heine@jacobs-university.de

TABLE S2: Optimized DFTB confinement parameters and valence shell configurations for Mo, W, and S.

Element	Valence shell	r_0/bohr	N
Mo	$4d^4$ $5s^2$ $5p^0$	4.3	11.6
W	$5d^4$ $6s^2$ $6p^0$	4.2	8.6
S	$3s^2$ $3p^4$ $3d^0$	3.9	4.6

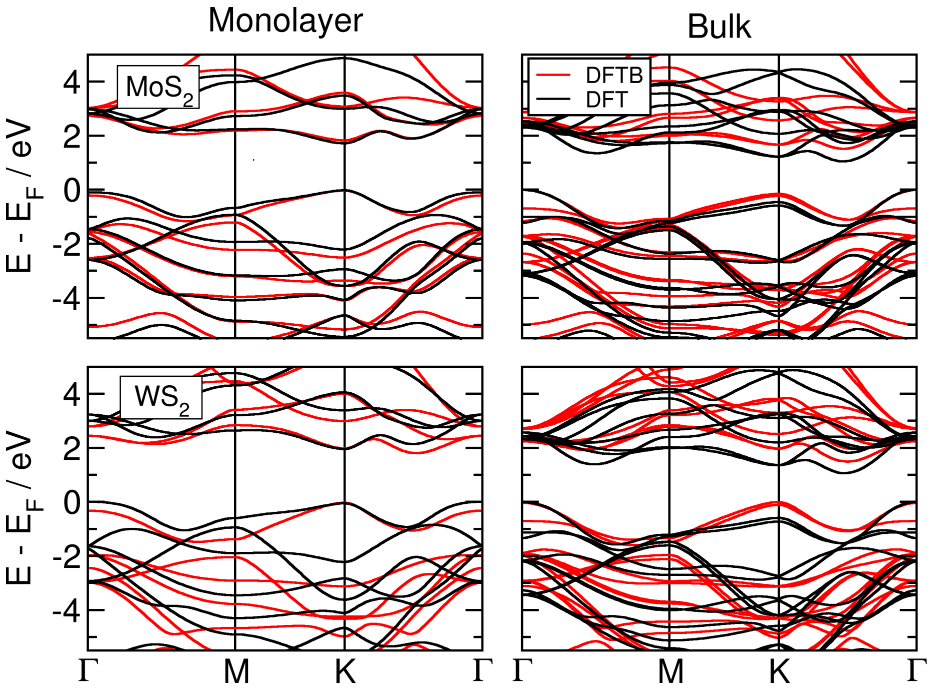

FIG. S1: DFT and DFTB electronic band structures calculated for bulk MoS_2 and WS_2 in bulk and monolayered forms. The Fermi level is shifted to the top of valence band.

such as the position of the gaps and general shape of the bands, are better described, as shown in Figure S1. For W and S we have used the standard electronic configurations. The final parameters chosen for the present study are summarized in Table S2.

[1] Perdew, J. P., Burke, K. & Ernzerhof, M. Generalized gradient approximation made simple. *Phys. Rev. Lett.* **77**, 3865 (1996).

[2] Philipsen, P. H. T. *et al.* Band2012. SCM, Theoretical Chemistry, Vrije Universiteit, Amsterdam, The Netherlands, http://www.scm.com (2012).

[3] Wiesenekker, G. & Baerends, E. J. Quadratic integration over the 3-dimensional brillouin-zone. *J. Phys.: Condens. Matter* **3**, 6721–6742 (1991).

[4] Velde, G. T. & Baerends, E. J. Precise density-functional method for periodic structures. *Phys. Rev. B* **44**, 7888–7903 (1991).
